# Transferrin-modified chitosan nanoparticles for targeted nose-to-brain delivery of proteins

**DOI:** 10.1007/s13346-022-01245-z

**Published:** 2022-10-07

**Authors:** Bettina Gabold, Friederike Adams, Sophie Brameyer, Kirsten Jung, Christian L. Ried, Thomas Merdan, Olivia M. Merkel

**Affiliations:** 1 Department of Pharmacy, Pharmaceutical Technology and Biopharmacy, Ludwig-Maximilians Universität München, 81377 Munich, Germany; 2 Institute of Polymer Chemistry, Chair of Macromolecular Materials and Fiber Chemistry, University of Stuttgart, Stuttgart, Germany; 3 Department of Biology I, Microbiology, Ludwig-Maximilians-Universität München, Martinsried, Germany; 4 Drug Product Development, AbbVie Deutschland GmbH, Ludwigshafen, Germany

**Keywords:** Chitosan nanoparticles, Transferrin receptor, Nose-to-brain, Brain delivery, Glioblastoma

## Abstract

Nose-to-brain delivery presents a promising alternative route compared to classical blood–brain barrier passage, especially for the delivery of high molecular weight drugs. In general, macromolecules are rapidly degraded in physiological environment. Therefore, nanoparticulate systems can be used to protect biomolecules from premature degradation. Furthermore, targeting ligands on the surface of nanoparticles are able to improve bioavailability by enhancing cellular uptake due to specific binding and longer residence time. In this work, transferrin-decorated chitosan nanoparticles are used to evaluate the passage of a model protein through the nasal epithelial barrier in vitro. It was demonstrated that strain-promoted azide–alkyne cycloaddition reaction can be utilized to attach a functional group to both transferrin and chitosan enabling a rapid covalent surface-conjugation under mild reaction conditions after chitosan nanoparticle preparation. The intactness of transferrin and its binding efficiency were confirmed via SDS-PAGE and SPR measurements. Resulting transferrin-decorated nanoparticles exhibited a size of about 110–150 nm with a positive surface potential. Nanoparticles with the highest amount of surface bound targeting ligand also displayed the highest cellular uptake into a human nasal epithelial cell line (RPMI 2650). In an air–liquid interface co-culture model with glioblastoma cells (U87), transferrin-decorated nanoparticles showed a faster passage through the epithelial cell layer as well as increased cellular uptake into glioblastoma cells. These findings demonstrate the beneficial characteristics of a specific targeting ligand. With this chemical and technological formulation concept, a variety of targeting ligands can be attached to the surface after nanoparticle formation while maintaining cargo integrity.

**Graphical abstract:**

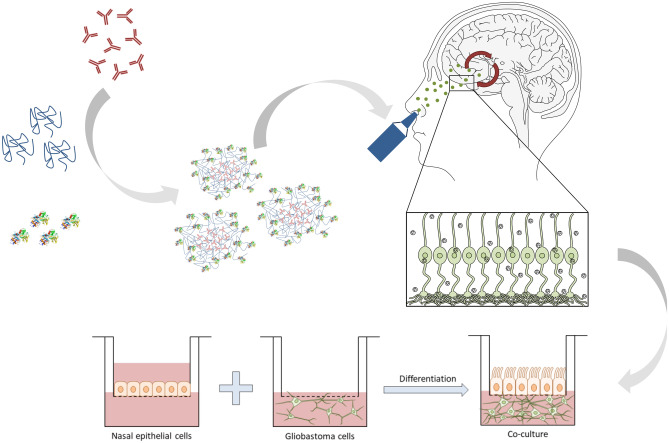

**Supplementary Information:**

The online version contains supplementary material available at 10.1007/s13346-022-01245-z.

## Introduction

Diseases of the central nervous system (CNS) such as Parkinson’s, Alzheimer’s, and brain cancer present a major health burden worldwide. A study from 2017 found that neurological disorders are the third most common cause of premature death and disability in the EU with prevalence being likely to increase due to the progressive ageing of the European population [1]. Adequate treatments for CNS disorders are challenging to develop regarding different aspects. The major obstacle for administered drugs is transit through the blood–brain barrier (BBB) or the blood-cerebrospinal fluid barrier (BCSFB) to reach the brain tissue [2]. Macromolecules such as proteins or nucleic acids, which represent an increasing fraction in the therapeutic landscape, are particularly limited in their ability to cross the BBB [3]. Although a variety of technological approaches have been investigated over the last decades trying to facilitate macromolecular drug transport into the brain, such accomplishments remained elusive [4–6]. Nose-to-brain (NtB) delivery presents an alternative route to reach the brain and, thus, gaining increasing interest over the past years. The advantages include non-invasiveness and rapid onset of action due to highly vascularized nasal mucosa. Furthermore, NtB is a direct route avoiding first-pass metabolism and circumventing the BBB [7]. It has been reported that drugs deposited on the olfactory region inside the nasal cavity can be delivered directly to brain tissue via olfactory or trigeminal nerve endings [8]. In particular, Reger et al. demonstrated the verbal memory improvement of Alzheimer disease patients after intranasally administering insulin without systemic side effects [9]. However, NtB delivery also has its limitations, especially for macromolecules sensitive to rapid degradation. When administered intranasally, many substances undergo fast elimination by enzymatic degradation, mucociliary clearance, and drainage to the lower part of the pharynx [10]. In this context, nanotechnology emerged to be a promising strategy for NtB delivery enhancement of therapeutic biomolecules [11, 12]. The cargo is protected against degrading effects of extracellular enzymes, and membrane efflux pumps may be bypassed [12]. Consequently, drug transport can be improved significantly after intranasal administration compared to administration of free drug [13, 14]. Furthermore, nanoparticle characteristics can be adjusted towards increased adherence to the mucus-covered epithelial cell layer promoting transport through tissue [15]. Chitosan nanoparticles (CS NPs) are one of these formulations, which are very attractive for NtB delivery due to chitosan’s biocompatibility, biodegradability, and mucoadhesion [16, 17]. In general, chitosan is the principal derivative of chitin, a structural component of the exoskeleton of Crustacea and insects, and is usually obtained by alkaline deacetylation [18]. Its sugar backbone is constituted of β-1,4-linked glucosamine with a low degree of *N*-acetylation. The cationic character together with the presence of reactive functional groups makes chitosan a very attractive polymer to use in controlled-release technologies [19–22]. CS NPs are already well established and can be prepared under mild conditions, e.g., via ionotropic gelation with a bridging agent in aqueous conditions [23]. Although, until now, chitosan nanoparticles have mostly been used for NtB delivery of small molecules, aforementioned advantages make them particularly suitable for sensitive cargos such as proteins or nucleic acids [24, 25]. 

In general, substances administered intranasally can be transported to the CNS via different pathways, namely via intracellular, paracellular, and transcellular mechanisms [26]. In the intracellular or transcellular olfactory nerve pathway, the substance is taken up by pinocytosis and endocytosis into the olfactory sensory neurons and transported alongside the axon to the olfactory bulb [27]. From there, the molecules can disperse throughout the brain. In the olfactory epithelial pathway, the substance is absorbed into the lamina propria before entering the CNS through the gaps surrounding the olfactory nerve tract [28, 29]. Substances can also be absorbed by lymphatic vessels or local blood vessels. However, most molecules are translocated through the perineural space via bulk flow to the subarachnoid space of the brain [27].

However, the olfactory region accounts for only about 5–10% of the total surface area inside the human nasal cavity [30–32]. The remaining area is covered by respiratory epithelia. Since olfactory nerves can only be found in the olfactory region, this is the main target site for NtB formulations. Because of the small and hard-to-reach target area located at the upper end of the nasal cavity, several targeting moieties have been studied to enhance the efficacy and specificity of NtB delivery, such as different lectins [14, 33, 34], lactoferrin [35–37], or cell-penetrating peptides [38–40]. Among these, lactoferrin is one of the most frequently used targeting moieties incorporated on the nanoparticle surface due to high expression levels of the lactoferrin receptor in neurons and brain endothelial cells [41]. Surface modification of PEG-PCL nanoparticles with lactoferrin led to an enhanced accumulation in the brain compared to non-modified nanoparticles according to an in vivo fluorescence imaging study by Liu et al. [35]. In this study, transferrin (Tf) was utilized as targeting ligand. On the one hand, the transferrin receptor is known to be expressed in human nasal cavity and, thus, it represents a promising targeting ligand [42, 43]. Additionally, proliferating cells and cells that have undergone malignant transformation, such as in glioblastoma multiforme, show an overexpression of transferrin receptor to regulate the amount of iron for metabolic needs. Therefore, a dual targeting effect can be achieved when using transferrin as surface ligand on nanoparticles [44, 45]. In general, transferrin belongs to the same protein family as lactoferrin and is an iron-binding glycoprotein of about 80 kDa, which is involved in cellular iron acquisition [46]. Cells recognize Tf via receptor-mediated endocytosis, while only the holo-form (diferric Tf with two binding sites occupied) and not the apo-form shows strong receptor binding [47]. Naturally, proteins exhibit many primary amine groups representing suitable binding sites for linker attachment. In general, surface modification of protein-loaded NP after preparation is not trivial. A very powerful tool for bioconjugation is represented by a set of biorthogonal chemical reactions referred to as “click” reactions, which are usually characterized by fast reaction speed, easy handling, versatility, regioselectivity, and high product yields [48, 49]. The most commonly used reaction is the copper(I)-catalyzed azide-terminal alkyne cycloaddition (CuAAC) reaction [50]. However, the use of this conjugation method has been restricted in biomedical applications due to toxicity concerns of the copper catalyst and the need for additional purification steps [51]. The strain-promoted azide–alkyne cycloaddition (SPAAC) reaction of azides with strained cyclooctynes presents a promising alternative because it proceeds without a copper catalyst. With this method, a functional group can be attached to both transferrin and chitosan enabling a rapid covalent surface-conjugation under mild reaction conditions after CS NP preparation.

Here we report the chemical modification, preparation, characterization, and in vitro evaluation of chitosan nanoparticles with human holo-transferrin as surface targeting ligand for a possible application in NtB delivery.

## Materials and methods

### Materials

Chitosan (5–20 mPa·s, 0.5% in 0.5% acetic acid at 20 °C, deacetylation degree of 85%) was purchased from TCI Chemical Industry Co., LTD (Tokyo, Japan). Ethyl 5-bromovalerate, 1-ethyl-3-(3-dimethylaminopropyl)carbodiimide (EDC), N-hydroxysuccinimide(NHS), DMSO, 2-(N-morpholino)ethanesulfonic acid (MES), human holo-transferrin expressed in rice, pentasodium tripolyphosphate (TPP), β-galactosidase (bGal) from *Aspergillus oryzae*, 2-nitrophenyl ß-D-galactopyranoside (ONPG), EMEM (Eagle’s Minimum Essential Medium), L-glutamine, penicillin/streptomycin, fetal bovine serum (FBS), human glioblastoma cell line U87, DMEM (Dulbecco’s Modified Eagle Medium), epidermal growth factor, insulin, hydrocortisone, trypsin–EDTA, and other routine chemicals were obtained from Sigma-Aldrich (Taufkirchen, Germany) and used as received. Dibenzyl cyclooctyne-N-hydroxysuccinimide-ester (DBCO-NHS-ester) and fluorescein (FAM) azide were acquired from Lumiprobe GmbH (Hannover, Germany). ATTO647N-NHS dye was bought from ATTO-TEC GmbH (Siegen, Germany). His-tagged transferrin receptor (TfR-His) was purchased from Sino Biological Inc. (Beijing, People’s Republic of China). Human nasal squamous carcinoma cell line RPMI 2650 was obtained from Cell Lines Services (Eppelheim, Germany) and human breast epithelial cells MCF-10A from ATCC (Manassas, USA). Air–liquid interface (ALI) differentiation medium was bought from Lonza (Basel, Switzerland). Anti-human CD71 (PE, clone OKT 9, eBioscience™) and anti-mouse IgG1 (PE, clone M1-14D12, eBioscience™) were purchased from Thermo Fisher Scientific (Waltham, MA, USA). Biacore His-capture kit was obtained from Cytiva (Marlborough, MA, USA).

### Modification of chitosan

The precursor for the click reaction, azide-modified chitosan was prepared according to literature [52]. In brief, an amidation of chitosan with 5-azidopentanoic acid in the presence of EDC/NHS was performed. For this reaction, 5-azidopentanoic acid was synthetized according to literature [53]. In a 25-mL round bottom flask, 2.50 mg of ethyl 5-bromovalerate (11.9 mmol, 1 equiv.) was dissolved in 6.25 mL DMSO and consecutively 3.07 g sodium azide (47.7 mmol, 4 equiv.) was added while stirring. The reaction mixture was stirred for 24 h at 100 °C. After cooling, the brown suspension was treated with 100 mL water. Then, the solution was extracted with ethyl ether (4 × 50 mL) and the combined organic phase was concentrated to approx. 30 mL in vacuo. This solution was diluted with 30 mL of 1 M NaOH_(aq)_ and the mixture was stirred overnight at room temperature. After washing the solution with ethyl ether (3 × 20 mL), it was acidified to pH 1 with HCl_(conc)_. The product was extracted with ethyl ether (3 × 20 mL). The combined organic phases were dried over MgSO_4_, filtered, and the solvent was removed in vacuo. 1.29 g of 5-azidopentanoic acid was obtained as a yellow liquid (yield 52 wt%). For the amidation of chitosan with 5-azidopentanoic acid, 0.50 g chitosan (2.73 mmol, 1.0 equiv.) and 0.39 g 5-azidopentanoic acid (2.73 mmol, 1.0 equiv.) were dissolved in 50 mL MES buffer and the reaction mixture was stirred for 5 h at room temperature. Then, the solution was degassed with nitrogen for 30 min. Afterwards, 1-ethyl-3-(3-dimethyl aminopropyl)carbodiimide (EDC, 1.57 g, 2.73 mmol, 1.0 equiv.) and *N*-hydroxysuccinimide (NHS, 2.83 g, 24.6 mmol, 9.0 equiv.) were gradually added to the flask within 20 min and left to react while stirring for 16 h. The polymer solution was dialyzed (MWCO 6–8 kDa) against distilled water for 6 days at 4 °C to remove impurities and freeze-dried afterwards. Azide-functionalized Chitosan (0.47 g, yield 53 wt%) was obtained as a slightly yellow powder.

### Modification of transferrin

Human holo-Tf expressed in rice was used for the modification with an alkyne group. For each batch, 30 mg Tf (0.38 µmol, 1.0 equiv.) was dissolved in 900 µL of 50 mM phosphate buffer pH 8. In another glass vial, 1.64 mg of dibenzyl cyclooctyne-*N*-hydroxysuccinimide-ester (DBCO-NHS-ester, 3.8 µmol, 10 equiv.) was dissolved in 100 µL DMSO and then added to the Tf solution. The reaction mixture was stirred overnight at 300 rpm at 4 °C. Afterwards, the conjugate was purified using a Sephadex G-25 pre-packed PD-10 column and freeze-dried subsequently. As product, 25.2 mg of alkyne-functionalized Tf (yield 79.5 wt%) was obtained as a slightly red powder. For the analysis of the conjugation reaction, IR spectra were recorded. Furthermore, the apparent weight of the protein and successful modification for use in click chemistry were evaluated by SDS-PAGE. In brief, fluorescein (FAM) azide was used as a fluorescent click-partner for the alkyne-modified Tf. Briefly, 10 µl of FAM azide (5 mg/mL, 0.075 mM, 3.75 equiv.) solution in DMSO and 100 µL sodium ascorbate solution (5 mM) were added to 1 mL of alkyne-modified Tf (1 mg/mL, 0.02 mM, 1.0 equiv.) in water while gently shaking the vial. The reaction mixture was incubated at room temperature in the dark for 2 h without shaking. For the verification of covalent linkage and intactness of protein, an SDS-PAGE was performed using a Novex WedgeWell 8–16% Tris–Glycine Gel (1.0 mm × 12 well). Unmodified holo-Tf was used as control. The gel was stained with Coomassie blue and analyzed with a ChemiDoc imager (Bio-Rad Laboratories, Inc., Feldkirchen, Germany). Subsequently, the gel was destained and analyzed again under UV-light to visualize fluorescent bands.

### Nanoparticle preparation

#### Preparation of chitosan nanoparticles

Chitosan nanoparticles were prepared via ionotropic gelation method according to a well-established and previously published protocol [23]. Briefly, 5 mg chitosan (CS) was dissolved in 5 mL of 25 mM acetate buffer pH 5.0 while stirring overnight. Pentasodium-tripolyphosphate (TPP), which has anionic character and, therefore, serves as a bridging agent in CS NP preparation, was dissolved in highly purified water with a final concentration of 1 mg/mL. Both solutions were filtered through a 0.22 µm mixed-cellulose-ester (MCE) filter. Subsequently, 2 mL of TPP solution were quickly added to the chitosan solution while stirring at 1000 rpm. The suspension turned slightly opaque, indicating successful nanoparticle formation, and was left stirring for 2 h. Nanoparticles were centrifuged on a 20-µL glycerol bed at 10,000 rpm and at 4 °C for 45 min. The supernatant was removed. For loaded nanoparticles, 2 mg of β-galactosidase (bGal) or ATTO647N-labeled β-galactosidase (ATTOGal) was added to the TPP solution before mixing with chitosan. Labeling of β-galactosidase with ATTO647N was performed according to the manufacturer’s protocol using a twofold molar excess of dye. Briefly, ATTO647N-NHS dye in DMSO was added to an aqueous protein solution at a concentration of 5 mg/mL and incubated for 1 h at room temperature. Afterwards, the protein–dye-conjugate was purified using a Sephadex G25 column.

#### Preparation of transferrin-decorated chitosan nanoparticles

For the preparation of transferrin-decorated chitosan nanoparticles, azide-modified CS was mixed in a 1:1 weight ratio with regular CS to a final concentration of 1 mg/mL in 25 mM acetate buffer pH 5.0 (CS mixture). Alkyne-modified Tf was dissolved in highly purified water at different concentrations (1, 2, 2.5, 4, and 5 mg/mL). CS mixture was filtered through a 0.45-µm MCE filter. TPP and bGal were dissolved in highly purified water at a concentration of 1 mg/mL each. After adding the bridging agent TPP and protein to the CS solution, the dispersion was stirred for 1 h before adding 1 mL of alkyne-modified Tf in the respective concentration. This reaction mixture was stirred for 1 h at 1000 rpm. Then, the nanoparticles were centrifuged at 10,000 × g for 45 min on a 20-µL glycerol bed, and the supernatant was removed.

### Nanoparticle characterization

#### Size and zeta potential determination

For evaluation of hydrodynamic diameter and polydispersity index (PDI), dynamic light scattering was used, and laser Doppler anemometry was applied for zeta potential analysis. One hundred microliters of NPs resuspended in highly purified water was added into a disposable micro-cuvette (Malvern Instruments, Malvern, UK) and size as well as PDI was determined with a Zetasizer Nano ZS (Malvern Instruments) at 173° backscatter angle performing 15 runs three times per sample. Viscosity of 0.88 mPa$\cdot$s and a refractive index of 1.33 were set for data analysis using the Zetasizer software. NPs were then diluted with 900 µL 10 mM NaCl, and 700 µL of NP suspension was transferred to a folded capillary cell (Malvern Instruments) to perform three zeta potential measurements per sample using the same device.

#### Encapsulation of model protein

For the determination of encapsulation efficiency, a modified version of β-galactosidase assay was conducted to assess enzymatic activity [54–56]. This analysis was performed indirectly and, thus, supernatants after centrifugation were used. Briefly, 8.33 µL of supernatant was added to 158.33 µL enzyme buffer containing 60 mM Na_2_HPO_4_, 60 mM NaH_2_PO_4_, 1 mM MgSO_4_, and 0.27% β-mercaptoethanol, in a transparent 96-well plate. For the blank reference, 167.66 µL of enzyme buffer were used. For generating a calibration curve, the same procedure as for samples with varying known concentrations of bGal was applied. The plate was equilibrated at 28 °C for 5 min in a plate reader (Tecan Group AG, Männedorf, Switzerland). Then, 33.3 µL of 2-nitrophenyl β-D-galactopyranoside (ONPG) with a concentration of 4 mg/mL in highly purified water was added to each well. The enzymatic reaction was measured for 10 min. The amount of encapsulated protein was determined by the following equation:

$$EE\;\left[\%\right]=\left(1-\frac{c_{\mathrm{free}\;\mathrm{bGal}}}{c_{\max.\;\mathrm{bGal}}}\right)\ast100\%.$$

#### Determination of bound transferrin

Nanoparticles were lyophilized with an Epsilon 2-6D LSCplus (Martin Christ Gefriertrocknungsanlagen GmbH, Osterode am Harz, Germany) under the following conditions: Primary drying at − 50 °C for 4 h and then secondary drying at − 20 °C for 42 h, 5 °C for 4 h, and 25 °C for 12 h under vacuum (0.09 mbar). Lyophilized samples were used for the analysis of Tf binding efficiency on nanoparticle surface. Inductively coupled plasma optical emission spectroscopy (ICP-OES) was used to determine the iron amount of surface bound Tf. Lyophilized modified Tf was used as reference. Ten milligrams of lyophilized nanoparticles was weighed into a glass vial, acidified with 0.5 mL HNO_3_ (69%) and 1.5 mL HCl (37%) and heated in a laboratory microwave at 185 °C for 30 min. Samples were measured using a Varian Vista RL CCD Simultaneous ICP-OES.

#### Transferrin receptor binding

The binding affinities of the prepared Tf-decorated CS NPs to His-tagged transferrin receptor (TfR-His) were investigated by surface plasmon resonance (SPR) spectroscopy. HBS buffer (10 mM HEPES pH 7.4, 150 mM NaCl, 0.005% (v/v) Tween 20) was used as running buffer for all measurements. Tf-decorated nanoparticles were prepared as described above, redispersed in HBS buffer, and hydrodynamic diameters were measured to ensure no alterations caused by the high salt conditions (Supplementary Fig. 2). For comparison, free human holo-Tf and alkyne modified holo-Tf were dissolved in HBS buffer. SPR assays were performed in a Biacore T200 device using CM5 Series S carboxymethyl dextran sensor chips coated with anti-His-Tag antibodies from the Biacore His-capture kit. Briefly, the chips were equilibrated with HBS buffer until the dextran matrix was swollen. Afterwards, two flow cells of the sensor chip were activated with a 1:1 mixture of N-ethyl-N-(3-dimethylaminopropyl)carbodiimide hydrochloride and N-hydroxysuccinimide according to the standard amine coupling protocol. A final concentration of 50 µg/mL anti-His-Tag antibody in 10 mM acetate buffer pH 4.5 was loaded onto both flow cells using a contact time of 420 s for gaining a density of approximately 10,000 resonance units (RU) on the surface. By injection of 1 M ethanolamine/HCl pH 8.0, free binding sites of the flow cells were saturated. Preparation of chip surfaces was carried out at a flow rate of 10 µL/min. For interaction analysis, TfR-His (30 ng) was captured onto one flow cell using a contact time of 60 s at a constant flow rate of 10 µL/min. This resulted in a capture density of approximately 200–300 RU of TfR-His. Nanoparticles with five different amounts of transferrin (1, 2, 2.5, 4, and 5 mg) were injected onto both flow cells using an association time of 240 s and a dissociation time of 600 s. The flow rate was kept constant at 30 µL/min. As control, a similar concentration of chitosan was injected. The chip was regenerated after each cycle by removing TfR-His completely from the surface using 10 mM glycine pH 1.5 for 60 s at a flow rate of 30 µL/min. To address the binding affinities of free human holo-Tf and modified Tf, SPR assays were performed as described above using these concentrations (1, 10, 25, 50, 2 × 100, 500, and 1000 nM) of free human holo-transferrin or alkyne modified holo-transferrin, respectively. All experiments were performed at 25 °C. Sensorgrams were recorded using the Biacore T200 Control software 2.0.2 and analyzed with the Biacore T200 Evaluation software 3.1. The surface of flow cell 1 was not coated with TfR-His and used to obtain blank sensorgrams for subtraction of the bulk refractive index background. The referenced sensorgrams were normalized to a baseline of 0. Peaks in the sensorgrams at the beginning and the end of the injection are due to the run-time difference between the flow cells for each chip. For the calculation of K_D_ values, steady-state affinity curves were used, and the kinetics were fitted assuming a 1:1 binding model using the Biacore T200 Evaluation Software 3.1.

### General cell culture

Human nasal squamous carcinoma cell line RPMI 2650 was cultured in EMEM (Eagle’s Minimum Essential Medium) supplemented with 1 mM sodium pyruvate, 0.1 mM non-essential amino acids, 2 mM L-glutamine, 1% penicillin/streptomycin, and 10% fetal bovine serum (FBS). Human glioblastoma cell line U87 was cultured in EMEM containing 1% penicillin/streptomycin and 10% FBS and human breast epithelial cells MCF-10A were cultured in DMEM (Dulbecco’s Modified Eagle Medium) supplemented with 10% FBS, 1% penicillin/streptomycin, 20 ng/ml epidermal growth factor, 10 μg/ml insulin, and 0.5 mg/ml hydrocortisone. The cell cultures were maintained at 37 °C in a > 95% humidified atmosphere of 5% CO_2_ in air with media changes on alternate days. Once 80–100% confluent, the cells were harvested with 0.25% trypsin–EDTA for further experiments.

### Co-culture model

A co-culture model of RPMI 2650 and U87 cells was established. First, RPMI 2650 cells were cultured at the air–liquid interface (ALI). Hence, they were seeded on PET Transwell™ membrane inserts (1 µm pore size, ∅ 0.33 cm, Corning, NY, USA) with a density of 4 × 10^5^ cells/cm^2^. Air-lift was performed after 24 h by removing the medium from the apical compartment and replacing the medium on the basolateral compartment with ALI differentiation medium. TEER values of RPMI 2650 cell layers were determined every 4–5 days to determine cell monolayer integrity using an epithelial voltohmmeter (EVOM2, WPI, Sarasota, FL, USA) with a chamber electrode (EndOhm, WPI). For the measurement, 700 µL of regular culture medium was added to the electrode chamber and 150 µL to the apical side compartment of the insert. The measured TEER values were corrected by subtracting the mean resistance of blank porous membranes. After 21 days of culture at 37 °C and 5% CO2, cells showed steady TEER values and were used for further experiments. After 20 days, U87 cells were seeded with a density of 5 × 10^4^ cells/well in a 24-well plate. On day 21, the Transwell™ insert containing RPMI 2650 cells at ALI was placed into the 24-well plate containing U87 cells. The medium was changed and consisted of 90% ALI medium and 10% U87 medium. The co-culture was incubated at 37 °C in a > 95% humidified atmosphere of 5% CO_2_ for 4 h before using it for experiments.

### Transferrin receptor expression

For validation, whether the used cell line is suitable for targeting with Tf decorated CS NPs, the endogenous transferrin receptor 1 (CD71) expression levels were determined for each cell line. Hereby, MCF-10A cells were used as negative control. In general, cells were harvested following trypsinization and resuspended in PBS at 10^5^ cells/mL in triplicate for CD71, isotype control, and unstained samples. After incubation with the respective PE-labeled antibodies, samples were washed twice with PBS, and the median fluorescence intensity (MFI) was quantified using an Attune Nxt Flow Cytometer (Thermo Fisher Scientific, Waltham, MA, USA) with a 488-nm excitation laser and a 574/26-nm emission filter. All cells were gated according to morphology based on forward/sideward scattering, and 10,000 events were evaluated per sample.

This experiment was repeated with RPMI 2650 cells cultured under ALI conditions to confirm the presence of CD71 at the ALI in comparison to regular liquid culture.

### Cellular uptake

ATTOGal-loaded CS NPs with different amounts of surface-bound Tf (1, 2, 2.5, 4, and 5 mg) were used for the analysis of cellular uptake. RPMI 2650 cells were seeded in a density of 5 × 10^4^ cells/well in a 24-well plate. After 24 h incubation, they were treated with the different NP samples at a concentration of 0.1 mg/mL. After additional 4 h, cells were harvested via trypsinization, washed twice with PBS before resuspension in 400 µL PBS containing 2 mM EDTA and analysis via flow cytometry using 637 nm excitation and 670/14 nm emission filter. All cells were gated according to morphology based on forward/sideward scattering, and 10,000 events were evaluated per sample.

For the evaluation of NP passage through an epithelial cell layer, cells in co-culture were treated with NP at a concentration of 0.1 mg/mL. Hereby, free ATTOGal was compared to ATTOGal-loaded CS NPs and Tf-decorated nanoparticles. One half of the samples was incubated at 37 °C and the other half at 4 °C to distinguish between actual uptake and adhesion to the cell surface. After 24 h, cells were harvested and washed three times before resuspension in 400 µL PBS containing 2 mM EDTA. Samples were analyzed via flow cytometry with 637 nm excitation and 670/14 nm emission filter. All cells were gated according to morphology based on forward/sideward scattering, and 10,000 events were evaluated per sample.

### Statistical analysis

All results are given as mean value ± standard deviation (SD) of three individual experiments unless stated otherwise. Statistical significance was investigated using one-way ANOVA and two-way ANOVA with Bonferroni’s and Tukey’s post hoc post-test. All statistical analysis was performed using GraphPad Prism version 9.2.0 for Windows (GraphPad Software, San Diego, CA, USA, www.graphpad.com).

## Results and discussion

Specific targeting of cells in the olfactory region with NPs is hypothesized to improve intranasal protein delivery to the brain, thereby offering a promising alternative to conventional delivery over BBB. The targeting ligand should be covalently linked and solely bound on the NP surface in order to obtain a defined morphology, keep particle size as small as possible, and protect the encapsulated cargo [57, 58]. For this purpose, both CS and Tf were chemically modified according to previously published protocols introducing functional groups capable of undergoing a Huisgen-type SPAAC reaction [52, 53, 59].

### Modification of chitosan and transferrin

**Fig. 1:**
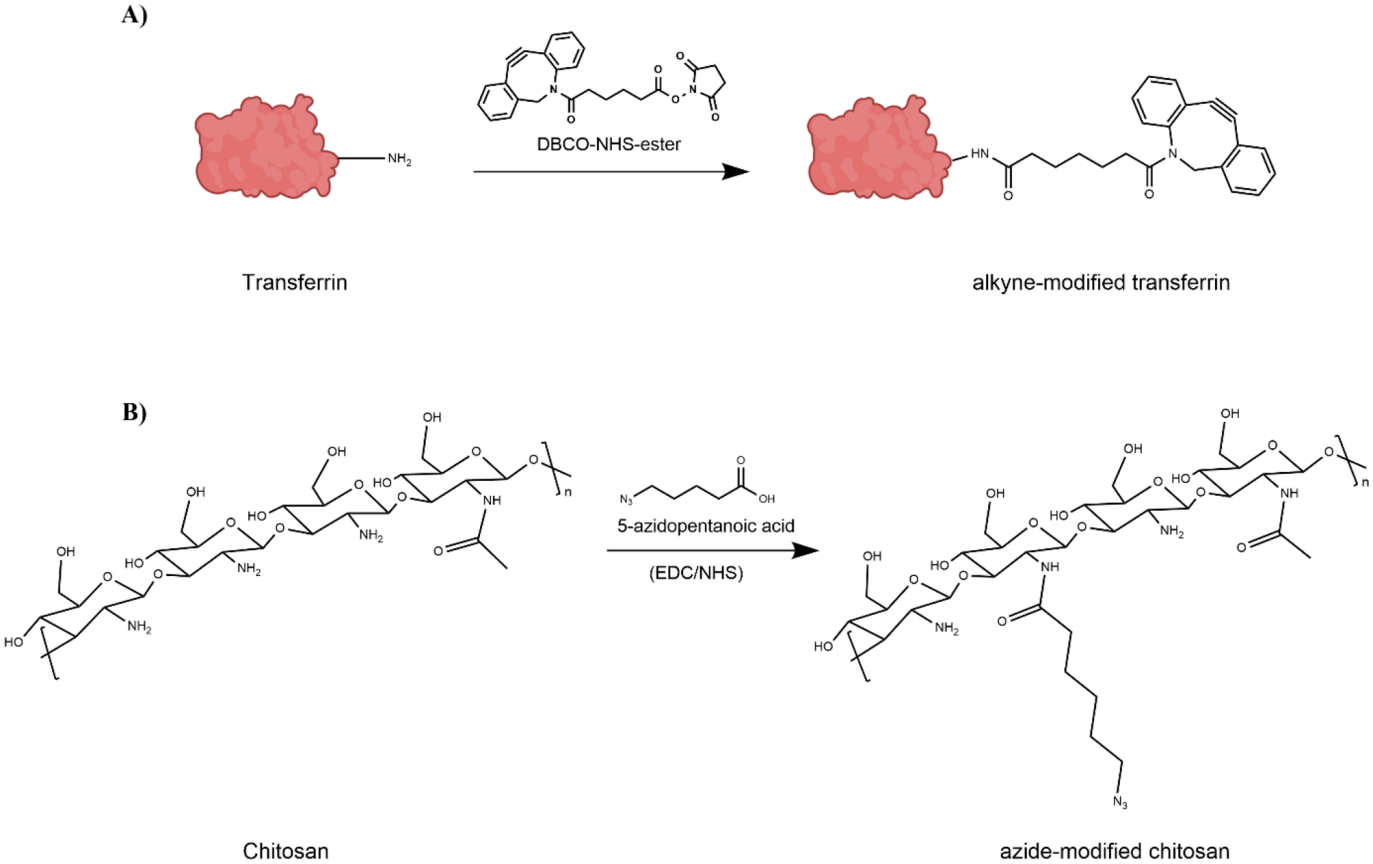
Chemical modification of transferrin and chitosan in order to introduce functional groups capable of undergoing click-reaction. **A** Modification of transferrin with DBCO-NHS-ester in the presence of EDC/NHS for generation of an alkyne functional group. **B** Modification of chitosan with 5-azidopentanoic acid for introducing azide functional groups

**Fig. 2:**
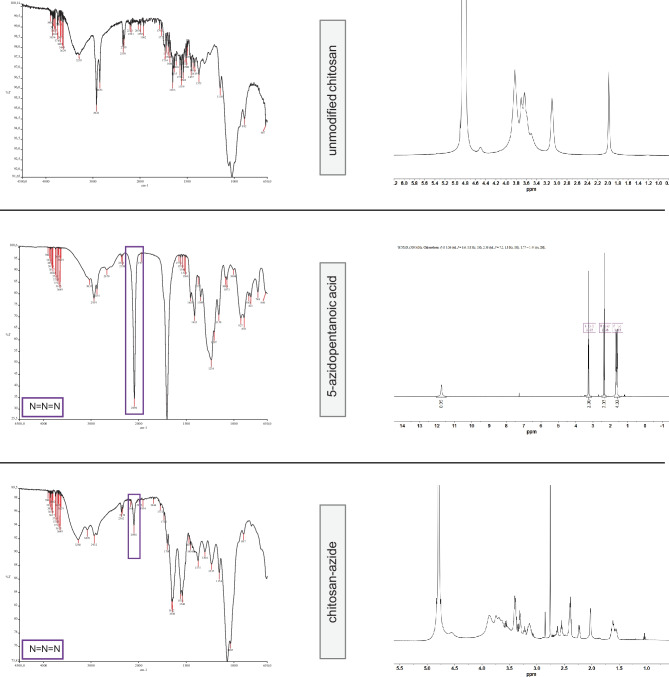
Analytics of chitosan after chemical modification. Right: IR spectra of unmodified chitosan, 5-azidopentanoic acid, and azide-chitosan. Left: ^1^H-NMR spectra of unmodified chitosan, 5-azidopentanoic acid, and azide-chitosan recorded in CDCl_3_

In general, Huisgen-type cycloaddtions are Cu^1^-catalyzed if a propargyl group is used [60]. For a copper-free “click” reaction between chitosan NPs and targeting ligand, the ring-strained alkyne derivative DBCO was chosen as functional group for transferrin (Supplementary Fig. 3) [61, 62]. By using this reaction type, it was possible to modify the NP surface after NP formation while preserving the cargo. For the preparation of Tf-decorated CS NPs, chemical modifications of both CS and Tf were performed (Fig. 1). The aim was to create a covalent, stable bond between the targeting ligand and the nanoparticles [62–64]. Therefore, azide-modified CS was prepared by amidation with 5-azidopentanoic acid in the presence of EDC/NHS. EDC can initiate the formation of an amide linkage between 5-azidopentanoic acid and CS by activating the carboxyl group and forming a reactive O-acylisourea intermediate, which spontaneously reacts with primary amines. However, this intermediate is not very stable in aqueous solutions. Thus, NHS is added and being activated by EDC, increasing stability of the reactive intermediate [65, 66]. This 2-step coupling allows for an efficient conjugation to primary amines under physiologic conditions. The IR spectra of unmodified CS, 5-azidopentanoic acid, and azide-modified CS reveal the appearance of an absorbance around a wave number of 2100 cm^−1^ in the azide-modified CS spectrum, which is characteristic for a terminal azide group (Fig. 2) [67]. These results are supported by ^1^H-NMR spectra shown in Fig. 2. The appearance of new signals and the splitting of protons suggest a coupling of 5-azidopentanoic acid to some of the amino repetition units in CS. These results indicate a successful CS modification.

**Fig. 3:**
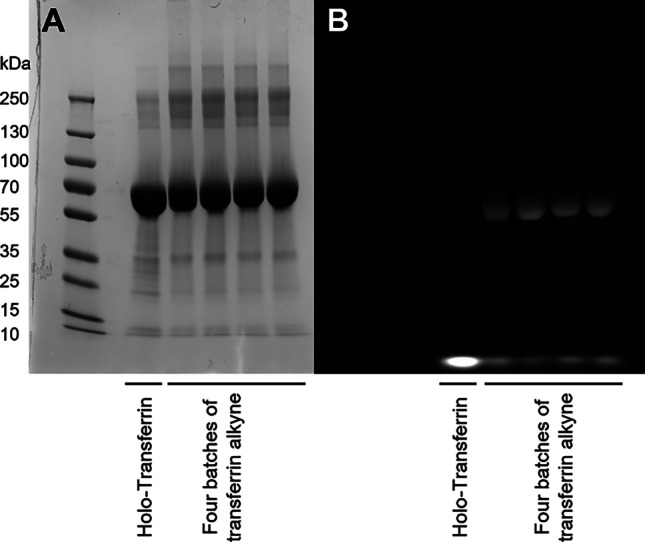
Alkyne-modified transferrin can be covalently modified by SPAAC as demonstrated by analysis via gel electrophoresis. Comparison of alkyne modified Tf and holo-Tf with Coomassie staining **A** and with gel visualization **B** under UV-light after click-reaction with FAM-azide

Tf was modified using DBCO-NHS-ester. This NHS-ester activated DBCO reacts with primary amines in physiologic to slightly alkaline conditions yielding stable amide bonds (Fig. 1) [59]. The resulting alkyne-modified Tf was first analyzed after click-reaction with FAM-azide by IR and SDS-PAGE. IR measurements revealed that the spectrum of unmodified holo-Tf corresponds to literature-reported Tf spectra (Supplementary Fig. 1) [68]. However, a difference between Tf and alkyne-modified Tf was not observed. Additionally, the characteristic resonances of DBCO at 1700 cm^−1^ and 754 cm^−1^ were too weak to be detected with this technique [69]. Therefore, another method was used to analyze whether modification of Tf was successful. First, modified Tf was reacted with FAM-azide, a fluorescent click-reaction partner, for 2 h at room temperature. Unmodified Tf was used as control. Subsequently, SDS-PAGE experiments were performed with this reaction mixture, and the protein bands were visualized using Coomassie blue. In the second step, UV light was used to detect the fluorescent click partner. The Coomassie staining in Fig. 3A shows that the protein bands of alkyne-Tf are similar to the ones of unmodified Tf, suggesting protein integrity after chemical alteration [63, 70]. Evidence for successful modification is shown in Fig. 3B. Due to the click-reaction with FAM-azide, the band of modified transferrin becomes visible under UV light together with the excess of fluorescent reaction partner at the bottom of the gel. Fluorescent bands show the same molecular weight as seen for the bands in Coomassie staining. This confirms that indeed it is modified-Tf that lights up due to the fluorescent label. Importantly, the fluorescence in the control sample can only be detected at the bottom of the gel, confirming that a conjugation did not take place with unmodified holo-Tf.

### Nanoparticle characterization

The advantages of attaching the targeting ligand after NP preparation are, on the one hand, the protection of the cargo due to reduced interactions with the ligand. On the other hand, the NP size is smaller since ligand molecules can solely be found on the NP surface. A NP size between 100 and 150 nm together with a positive surface potential is stated to be suitable characteristics for nose-to-brain delivery [71, 72]. As mucopolysaccharides are negatively charged, the positive NP surface charge is responsible for their mucoadhesive effect [73, 74]. Hence, after nanoparticle preparation via ionotropic gelation [23], hydrodynamic diameter, polydispersity index, and zeta potential were determined (Table 1). Chitosan nanoparticles without targeting ligand showed a size of about 120 nm, while an increase in size and also PDI was observed for Tf-decorated CS NPs. This is attributed to the Tf molecules bound on the NP surface having a molecular weight of about 80 kDa each and therefore increasing the hydrodynamic diameter of CS NPs. However, these Tf CS NPs showed higher PDI values suggesting a less homogenous sample. A possible explanation is the NP preparation via ionotropic gelation method in which it is not possible to exactly control the NP formation. As a result, there are different numbers of free Tf binding sites on the surface leading to a non-homogenous targeting ligand distribution. Additionally, Tf-decorated NPs exhibited a decreased zeta potential. It has been reported that Tf shields the positive surface charge of NPs, indicating successful attachment of targeting ligand on the NP surface [75]. Furthermore, the encapsulation efficiency of bGal was determined by an enzymatic assay as described above. CS NPs without ligand tend to exhibit a slightly higher encapsulation efficiency than Tf-decorated NPs which may be attributed to the number of overall charges in the CS molecule being decreased after chemical modification. Yet, within this sample size, this trend is not significant. Notably, an increase in added Tf amount does not have a significant impact on neither NP size or zeta-potential nor encapsulation efficiency. However, PDI values tend to increase with higher Tf concentrations suggesting lower sample homogeneity. This may originate from different amounts of Tf of NP surface, which are unevenly distributed.

**Table 1:** Evaluation of physicochemical characteristics of chitosan NPs without surface ligand and transferrin decorated chitosan NPs measuring hydrodynamic diameter, ζ potential, and encapsulation efficiency of bGal (values are given in mean ± SD.; *n* = 3)

**Transferrin conc. [mg/mL]**	**Size [nm]**	**PDI**	**ζ potential [mV]**	**Encapsulation efficiency [%]**
**0**	120 ± 11	0.194 ± 0.023	24.1 ± 2.9	63 ± 8
**1**	126 ± 14	0.211 ± 0.067	18.9 ± 2.1	54 ± 7
**2**	132 ± 16	0.222 ± 0.081	17.0 ± 2.7	54 ± 7
**2.5**	131 ± 21	0.235 ± 0.056	16.4 ± 1.4	55 ± 11
**4**	140 ± 17	0.227 ± 0.045	15.7 ± 1.6	51 ± 4
**5**	142 ± 23	0.251 ± 0.091	16.4 ± 2.2	47 ± 14

**Fig. 4:**
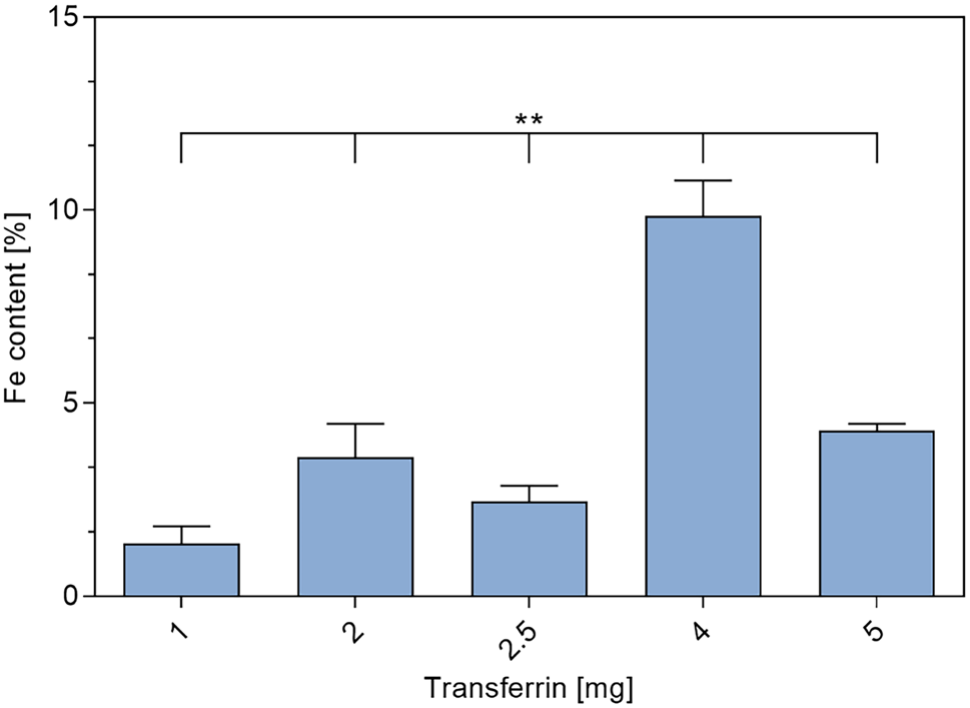
Iron content of transferrin-decorated chitosan nanoparticles using different amounts of transferrin in NP preparation (data points mean ± SD; *n* = 3; one-way ANOVA; one asterisk “*,” *p* < 0.05; two asterisks “**,” *p* < 0.01; three asterisks “***,” *p* < 0.005)

For the determination of Tf amount bound on the NP surface, the samples were analyzed using ICP-OES. The iron content of NP samples was detected and compared to free modified holo-Tf. The amount of surface-bound Tf was calculated with respect to the initially utilized amount of Tf during NP formation. Figure 4 shows different amounts of initially utilized Tf with their respective iron content after NP preparation. The analysis revealed that a 1:1 ratio between modified CS and modified Tf (corresponding to 2.5 mg of Tf in Fig. 4) leads to a binding efficiency of less than 5%. After optimizing the ratio to 1:1.6 (corresponding to 4 mg of Tf in Fig. 4), binding efficiency was improved to about 10%. Furthermore, there is no linear correlation since using an excess amount of Tf did not lead to clearly increasing surface binding.

**Fig. 5:**
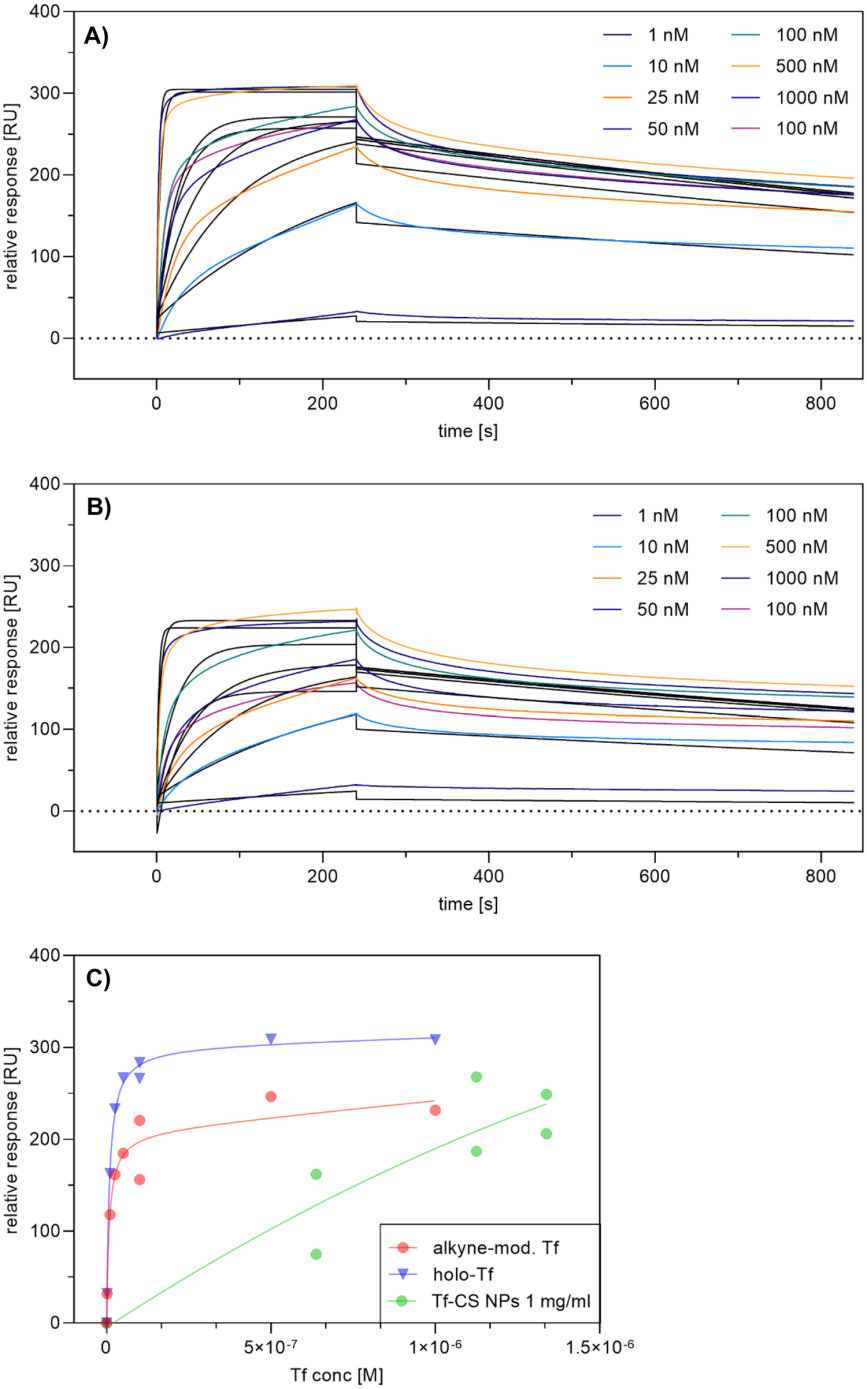
Binding of free holo-transferrin, free modified holo-transferrin, and transferrin-decorated CS NPs was analyzed by SPR spectroscopy. The transferrin receptor (TfR) was captured via His-tag onto a CM5 sensor chip coated with anti-His antibody, and different concentrations of analytes were tested. **A** Human holo-transferrin in different concentrations (1–1000 nM). **B** Alkyne modified holo-transferrin in different concentrations. **C** CS NPs with different surface-bound transferrin amounts (1, 2, 2.5, 4, and 5 mg/mL). The plots are representatives of three independently performed experiments. Black lines in panels **A** and **B** represent the fit of a 1:1 binding model (RU, response unit)

SPR measurements were used to determine binding affinity and kinetics of the interaction between plain and Tf-decorated CS NPs, respectively, with the transferrin receptor. Therefore, the His-tagged TfR was captured onto a sensor chip with anti-His-Tag antibodies on the surface before adding the NP samples. As positive control, free holo-Tf and free holo-Tf alkyne were injected in similar concentrations. A clear overall binding affinity of free holo-Tf to the TfR was detected with a K_D_ value of 1.40 nM, which is in agreement with the published K_D_ values in the range of 1–3 nM (Fig. 5A) [76–79]. Furthermore, free holo-Tf binding was characterized by an association rate of k_a_ = 2.88 $\cdot$ 10^5^ /M_d_ = 5.474 $\cdot$ 10^−4^ s^−1^. The binding affinity of alkyne transferrin seems to be similar with an overall K_D_ value of 1.47 $\cdot$ 10^−9^ M, an association rate of k_a_ = 3.84 $\cdot$ 10^5^, and a dissociation rate of k_d_ = 5.66 $\cdot$ 10^−4^ s^−1^ (Fig. 5B). These findings confirm that chemical modification of Tf does not impair the binding behavior to TfR. Together with SDS-PAGE results, SPR measurements confirm the structural integrity of alkyne-modified Tf. For comparison of NPs with different amounts of Tf immobilized on the surface, the samples were injected onto the biosensor chip one after the other keeping a constant concentration of 1 mg/mL Tf. Differences in binding behavior were evident, where an increased interaction was observed with increasing surface amount of Tf per NP. Although binding affinities of NPs to the TfR could not be determined due to the adhesiveness of CS to the biosensor chip, a comparison between different amounts of surface-bound Tf revealed consistency with ICP-OES measurements. The sample with the highest degree of Tf-modification also showed the highest receptor-binding response. In general, NP samples did not reach saturation but exhibited a linear correlation curve suggesting that the observed binding is not specific but still evident (Fig. 5C).

### Transferrin receptor expression

**Fig. 6:**
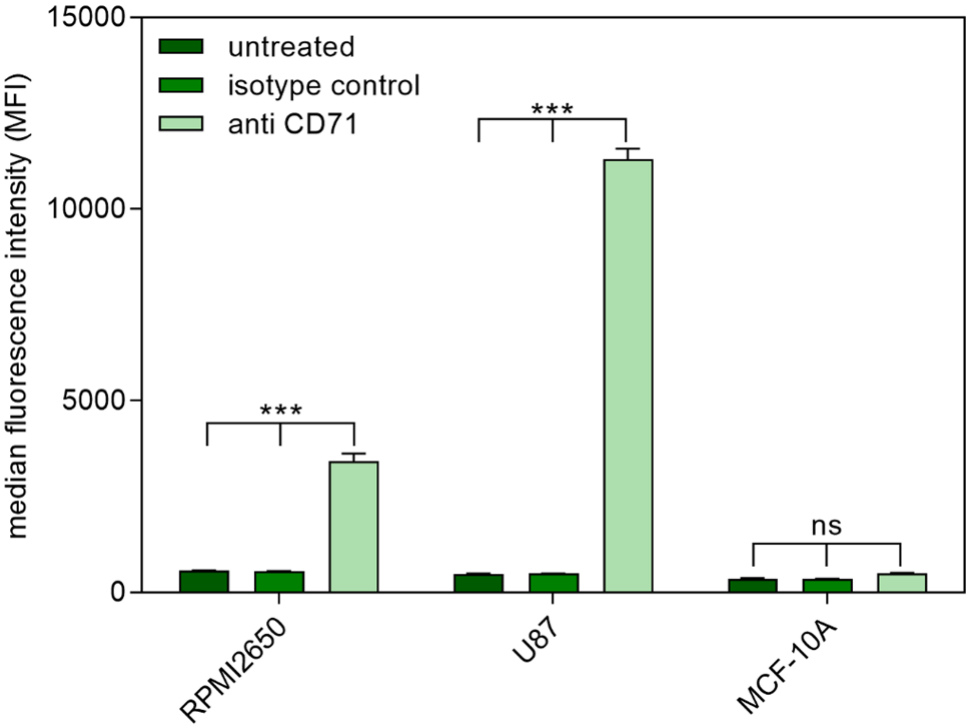
Transferrin receptor (CD71) expression of RPMI 2650 cells, U87 cells, and MCF-10A cells in liquid culture as measured by flow cytometry and compared to an isotype control and as well as untreated cells. Results are presented as median fluorescence intensity (data points indicate mean ± SD, *n* = 3; two-way ANOVA with Bonferroni’s post-test; ns, not significant; one asterisk “*,” *p* < 0.05; two asterisks “**,” *p* < 0.01; three asterisks “***,” *p* < 0.005)

**Fig. 7:**
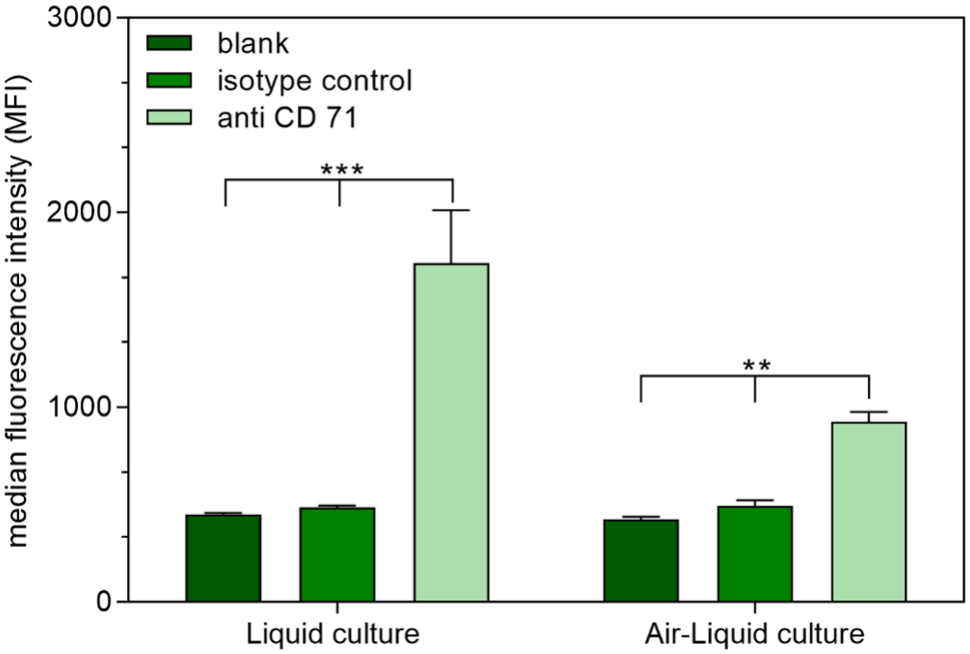
Transferrin receptor expression of RPMI 2650 cells cultured at ALI as measured by flow cytometry and compared to an isotype control as well as untreated cells. Results are presented as median fluorescence intensity (data points indicate mean ± SD, *n* = 3; two-way ANOVA with Tukey’s post-test; one asterisk “*,” *p* < 0.05; two one asterisk “**,” *p* < 0.01; one asterisk “***,” *p* < 0.005)

Although there is already evidence in literature that TfR is expressed in both cell lines used to mimic NtB delivery (RPMI 2650 and U87 cells), handling, passage number, and also cell line source might have an influence on cellular quality and characteristics [80, 81]. Therefore, TfR expression was examined by comparison with a breast epithelial cell line expressing too low levels of TfR for detection via flow cytometry [82]. Therefore, RPMI 2650 cells, U87 cells, and MCF-10A cells (negative control) were incubated with fluorescently labeled anti-CD71 antibody and an IgG isotype control antibody and analyzed via flow cytometry (Fig. 6). As expected, media fluorescence intensity (MFI) values of epithelial breast cancer cell line MCF-10A did not differ between cells incubated with anti-CD71 or isotype control, suggesting no detectable TfR expression. Both, U87 and RPMI 2650 cells, showed a significant TfR expression on the cell surface. Additionally, the expression level of U87 exceeded the one of RPMI 2650. Apart from more intricate techniques such as microfluidic 3D cell culture, air–liquid interface culture models of the respiratory tract are currently the most promising in vitro approach to mimic in vivo conditions in rather high-throughput experiments [83–85]. The most important characteristic of this model is that cells are cultured on a supporting membrane, so that the apical side is exposed to air while the basolateral side is in contact with nourishing medium. For the respiratory tract, this configuration grants cell differentiation towards a mucociliary phenotype resulting, for example, in the formation of tight junctions and the production of cilia and mucus [86–88]. RPMI 2650 cells are known to change morphology when cultured at ALI [89]. Therefore, their ability to retain CD71 expression levels at ALI after 21 days of culture was examined. The results confirm that after ALI cultivation, the cells still express TfR on the cell surface (Fig. 7). However, the expression levels are lower than in liquid culture, but still significantly increased compared to the isotype control and untreated sample. Due to the change in cell morphology at ALI, the apical and the basolateral side differ considerably from each other. We hypothesize that the TfR might only be expressed on the apical side leading to decreased TfR expression levels. As an overall result, the chosen cell lines are suitable for cellular uptake experiments with Tf as targeting ligand in liquid as well as ALI culture.

### Cellular uptake

In general, cationic materials such as CS can non-specifically interact with negatively charged cell membranes provoking adsorptive endocytosis [90, 91]. This non-targeted uptake could potentially interfere with the desired uptake into the olfactory region of the nasal cavity. It has already been demonstrated that cellular uptake is strongly dependent on surface ligand density [92]. Therefore, cellular uptake of Tf-decorated, ATTOGal-loaded nanoparticles in liquid culture of RPMI 2650 cells was evaluated via flow cytometry. Up to now, the RPMI 2650 cell line is the only cell line used as a nasal model [89]. However, it does not mimic nasal epithelium completely and further studies with excised nasal mucosa should be performed to accurately clarify the exact molecular mechanisms of NP uptake. Nonetheless, this cell line is widely used as tool for preclinical screening studies for nasal epithelial passage of different molecules and NPs [93]. The cells are fast and easy to culture and yield reproducible results [89]. ALI culture of RPMI 2650 cells is suitable to assess general passage through epithelial cell layers exhibiting tight junctions and important ABC-transporters [86, 88].Therefore, this cell line was used in this work to assess the differences between simple CS NPs and Tf-decorated NPs.

**Fig. 8:**
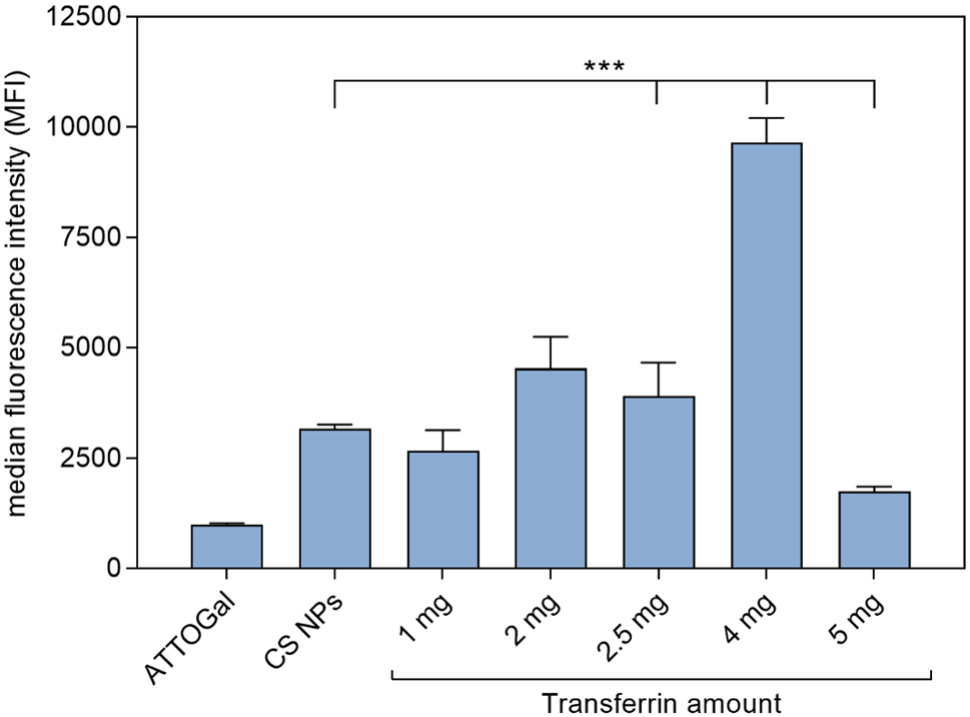
Cellular uptake of ATTOGal loaded transferrin-decorated CS NPs in liquid culture of RPMI 2650 nasal epithelial cells as measured by flow cytometry and compared to CS NPs without transferrin as well as free ATTOGal. Results are presented as median fluorescence intensity (data points indicate mean ± SD, *n* = 3; two-way ANOVA with Tukey’s post-test; one asterisk “*,” *p* < 0.05; two asterisks “**,” *p* < 0.01; three asterisks “***,” *p* < 0.005)

In the cellular uptake experiment, free ATTOGal and unmodified CS NPs were compared to NP samples with different amounts of Tf on the surface (Fig. 8). All NP samples exhibited an enhanced cellular uptake compared to free bGal. Furthermore, addition of Tf-decorated CS NPs with the highest degree of Tf modification showed the biggest increase in cellular uptake. Although there is no evident correlation between utilized Tf amount and cellular uptake, there is a trend of uptake enhancement with increasing amounts of bound Tf. Taking into account the results from ICP-OES measurements (Fig. 4), it is evident that the cellular uptake corresponds to the amount of Tf bound on the NP surface. This also explains the decrease of cellular uptake for the sample with 5 mg Tf compared to NPs prepared with 4 mg Tf. In the former NP formulation, less surface-bound Tf is available in comparison to the latter although the initially used Tf feed was higher. Conclusively, NPs with 4 mg of Tf, which exhibited the highest overall amount of surface-bound Tf, showed the best cellular uptake.

**Fig. 9:**
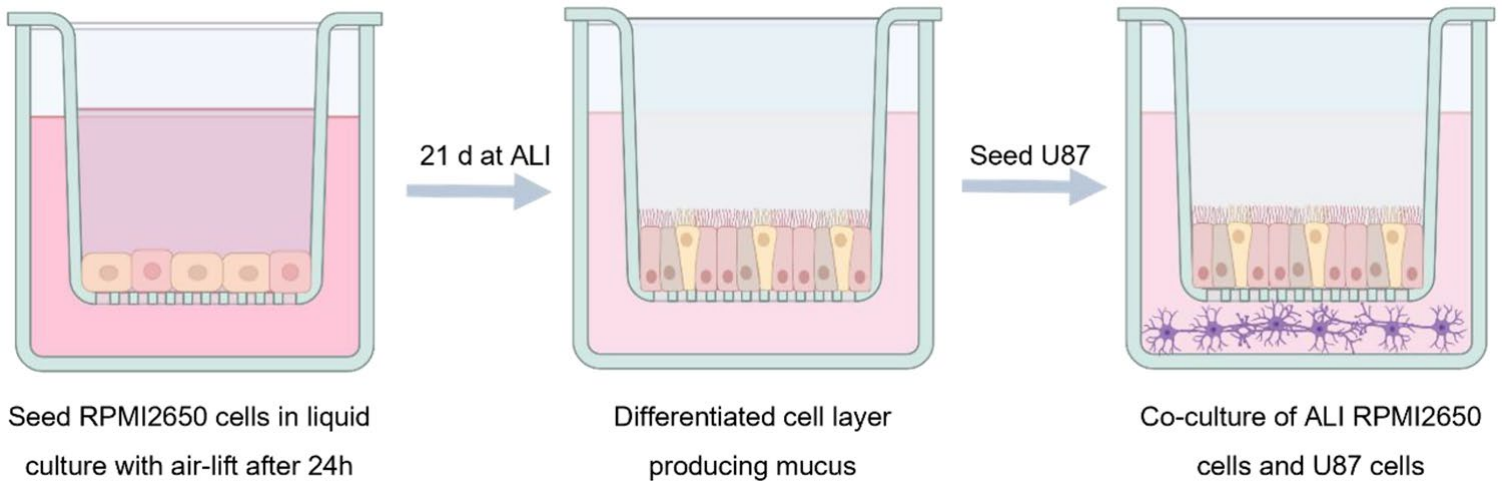
Schematic illustration of co-culture preparation using nasal epithelial cell line RPMI 2650 and glioblastoma cell line U87

**Fig. 10:**
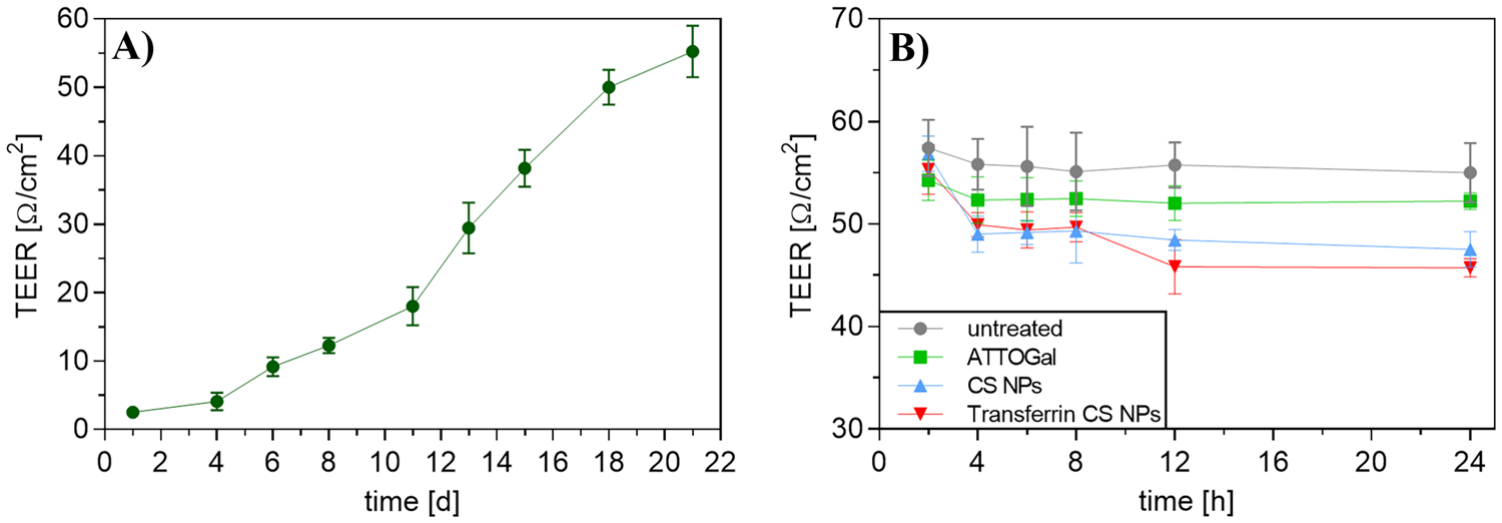
TEER measurements of RPMI 2650 ALI cell cultures for the assessment of cell monolayer integrity. **A** Development of TEER values of ALI cells during 21 days of culture period. **B** TEER values of ALI cells treated with ATTOGal, CS NPs or Transferrin CS NPs, respectively. Results are presented as mean ± SD (**A** *n* = 12; **B** *n* = 3)

Hence, for cellular uptake into the co-culture model, CS NPs with 4 mg of modified Tf were prepared. This co-culture was used to evaluate not only the passage through an epithelial cell layer at the air–liquid interface but also the subsequent uptake into cells found in brain tissue. Therefore, human nasal epithelial cells RPMI 2650 were cultured on a Transwell™ membrane insert and incubated for 21 days until the TEER values were > 50 Ω cm^2^ (Fig. 10A) [89]. This TEER value guarantees cell layer integrity and tight junction formation suggesting full cell differentiation [86, 87, 94]. Consecutively, the insert holding the differentiated cells was added to a 24-well plate containing human glioblastoma cells (U87) cells at the bottom of the well (Fig. 9).

This co-culture model was then treated with NP samples and incubated for 24 h. TEER was measured at different time points throughout the cellular uptake experiment to ensure cell monolayer integrity. The free ATTOGal sample showed a slight, statistically insignificant decrease after 4 h and remained constant for the rest of the uptake experiment. As chitosan is known for being able to open tight junctions to a certain extent, an initial decrease in TEER of about 7 Ω/cm^2^ was observed for both NP samples [95]. Afterwards, TEER values remained constant suggesting an integer cell layer with no signs of cytotoxicity (Fig. 10B).

**Fig. 11:**
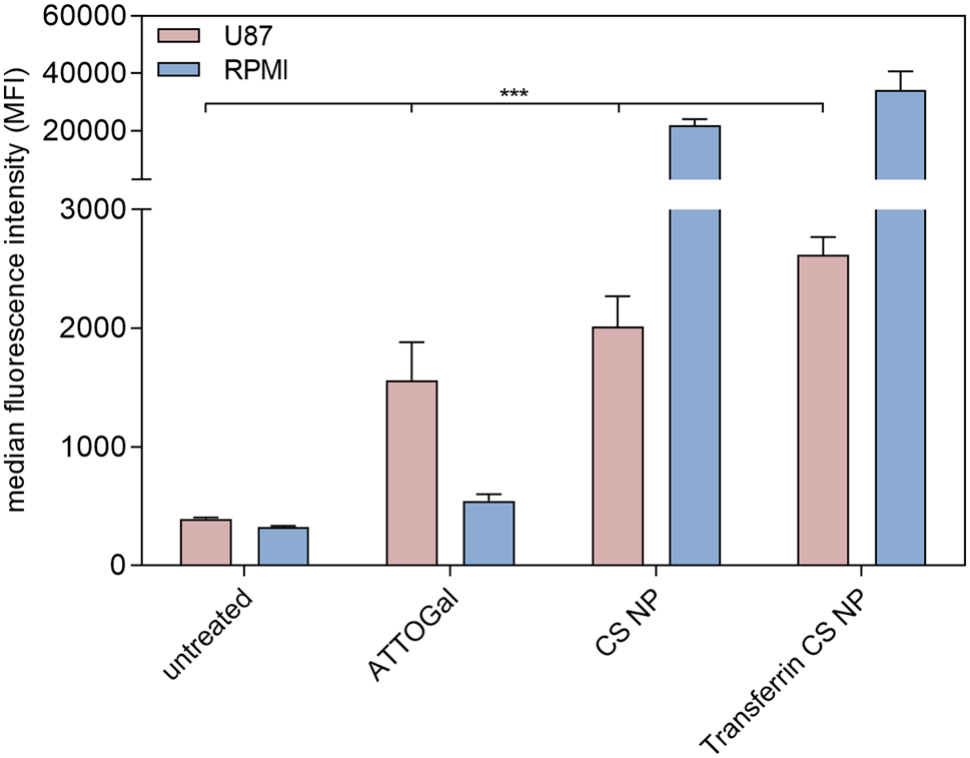
Cellular uptake of ATTOGal, loaded transferrin decorated CS NPs into a co-culture of U87 glioblastoma cells and ALI RPMI 2650 nasal epithelial cells as measured by flow cytometry and compared to ATTOGal loaded CS NPs without transferrin and free ATTOGal. Results are presented as median fluorescence intensity (datapoints indicate mean ± SD, *n* = 3; two-way ANOVA with Bonferroni’s post-test; one asterisk “*,” *p* < 0.05; two asterisks “**,” *p* < 0.01; three asterisks “***,” *p* < 0.005)

Figure 11 shows the MFIs of both cell lines. Free ATTOGal was compared to ATTOGal-loaded CS NPs and Tf-decorated NPs. At 24-h post treatment, the free protein can be mainly found in the U87 cells suggesting a rapid passage through the epithelial cell layer. Encapsulation of ATTOGal into CS NPs also led to an efficient uptake into U87 cells. However, after 24 h, a significant amount of NPs was still found in the RPMI 2650 cell layer. This may be attributed to different reasons. The presence of mucus slows down NPs more than the much smaller protein. Additionally, receptor-mediated transcytosis is a rather slow process, so that a considerable amount of NPs has not yet passed the epithelial layer after 24 h. Attaching Tf on the NP surface seemed to improve the cellular uptake into both types of cells. However, this trend was not significant. The amount of Tf in relative comparison to CS is only about 10% resulting in a rather low number of targeting ligands on the NP surface. This number can be increased by addition of more modified CS in the NP preparation process. However, parameters have to be optimized for every new blend ratio since particle size, PDI, and zeta-potential can vary drastically (data not shown). Another hypothesis explaining the low cellular uptake of Tf-decorated CS NPs is that of valency. We have recently described how monovalent ligands cannot fully outcompete multivalent ones [96]. Yet, it was also described that for Tf-mediated endocytosis size is more important than multivalency [97]. The reason is that clathrin-mediated uptake and transcytosis, which is the predominant pathway of internalization for Tf, are limited by the size of natural clathrin-coated pits (~ 100–150 nm) [98]. Hence, adjusting the formulation regarding both parameters will presumably result in improved uptake behavior [99]. Nonetheless, it was successfully shown that Tf-decorated CS NPs are able to cross the epithelial barrier in vitro, retain their targeting function, and be taken up into therapeutically relevant target cells. Despite the results obtained in this study, further evaluation on the best transport pathway for NtB delivery has to be conducted, in particular regarding transport time and delivery of therapeutic concentrations.

## Conclusion

Targeting the brain still is an especially challenging task due to the presence of the BBB [100]. Here, nose-to-brain delivery presents a promising alternative for direct drug delivery to brain tissue by circumventing the BBB, especially for biomolecules such as proteins or nucleic acids, which are recently growing in number for therapeutic use [29, 100–102]. Among other materials, CS NPs have shown great potential for intranasal administration, mainly because of their mucoadhesive and tight junction-opening properties [12, 103, 104]. For improved and specific drug delivery, NPs are often decorated with targeting ligands facilitating cell entry [105]. In this work, the aim was to create a concept for protein-loaded CS NPs with a surface bound targeting ligand that can easily be exchanged according to the therapeutic need. For this study, Tf was chosen as proof-of-concept targeting ligand since it is already well studied and the transferrin receptor can be exploited as target for nose-to-brain delivery. After introducing and analyzing functional groups to enable a versatile copper-free click chemistry, we have successfully prepared Tf-decorated CS NPs. With this method, a variety of targeting ligands can be attached to the surface after NP formation leading to smaller and more defined formulations. We also demonstrated that these NPs were specifically internalized by cells expressing TfR via receptor-mediated endocytosis. Hereby, we were able to tune the amount of surface ligands resulting in different binding behaviors. Furthermore, this system might be relevant in nose-to-brain delivery of proteins by passage through a mucus-producing epithelial cell layer with consecutive uptake into potential target cells. However, this hypothesis has to be proven in further studies, potentially on excised nasal mucosa, together with a more detailed analysis of which transport mechanism can be favorable for NtB delivery. Nonetheless, Tf-decorated CS NPs produced via copper-free click reaction offer an auspicious platform with easily interchangeable surface ligands for the targeted delivery of macromolecules.

## Supplementary Information

Below is the link to the electronic supplementary material.

Supplementary file1 (TIF 34 KB)

Supplementary file2 (TIF 198 KB)

Supplementary file3 (TIF 109 KB)

Supplementary file4 (PPTX 278 KB)

## Data Availability

The datasets generated during and/or analyzed during the current study are available from the corresponding author on reasonable request.
