# Supplementary figures and images for: Transferrin-modified chitosan nanoparticles for targeted nose-to-brain delivery of proteins
Source: Drug Deliv Transl Res. 2022 Oct 7;13(3):822–38. doi: 10.1007/s13346-022-01245-z (PMC9892103; doi:10.1007/s13346-022-01245-z)

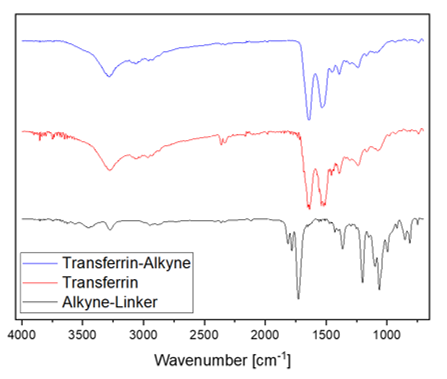

Supplement: Supplementary file 1 — Supplementary file1 (TIF 34 KB) [file 13346_2022_1245_MOESM1_ESM.tif]

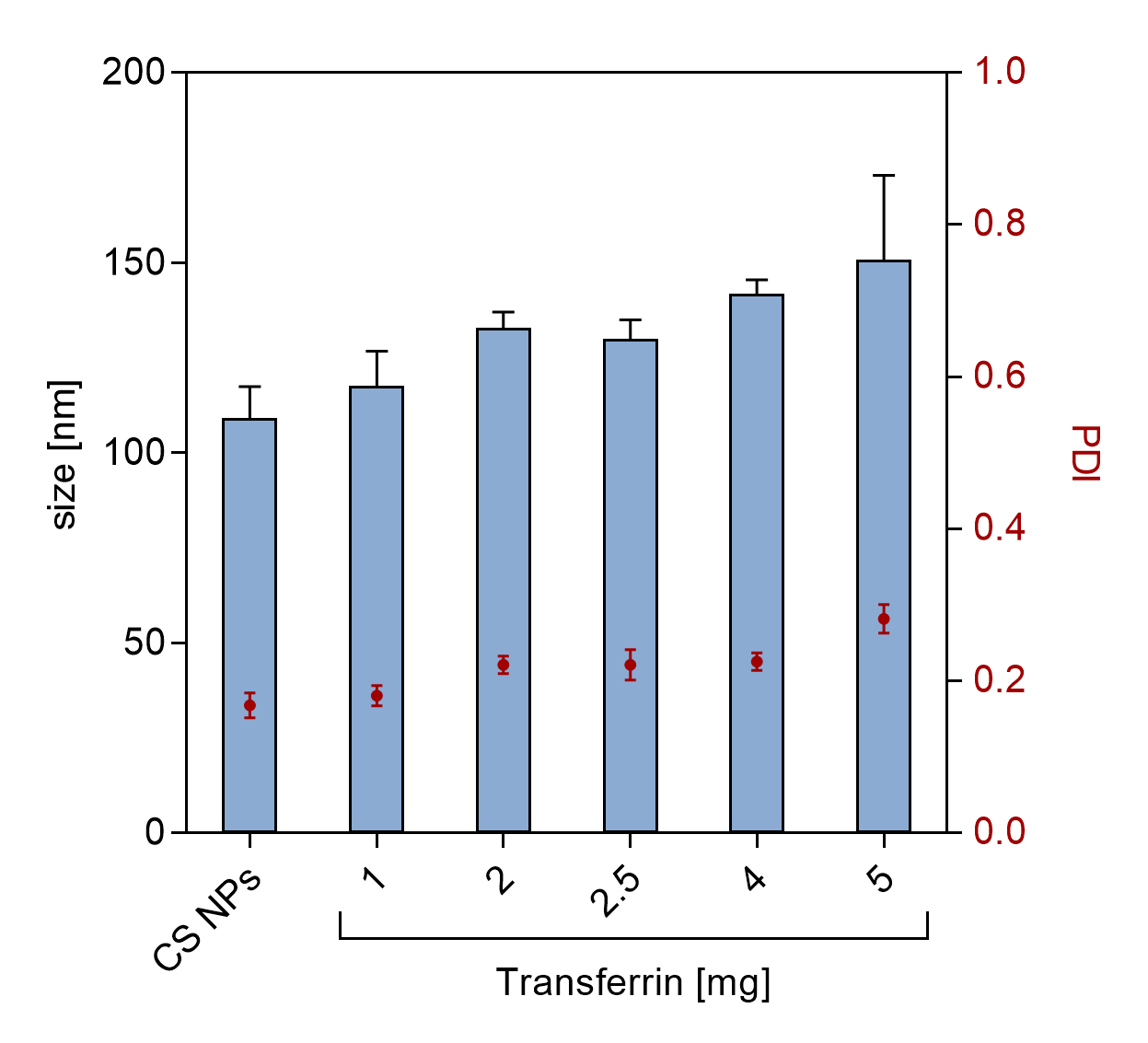

Supplement: Supplementary file 2 — Supplementary file2 (TIF 198 KB) [file 13346_2022_1245_MOESM2_ESM.tif]

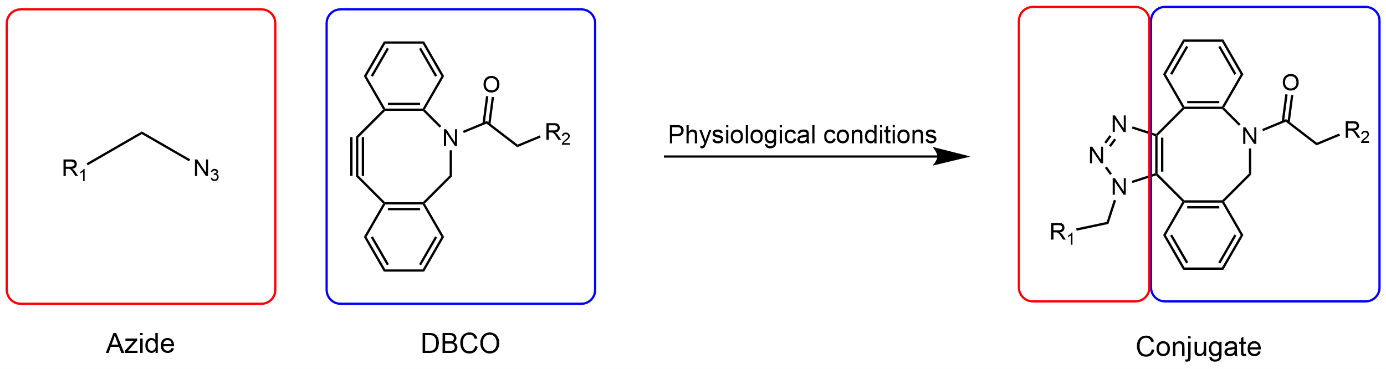

Supplement: Supplementary file 3 — Supplementary file3 (TIF 109 KB) [file 13346_2022_1245_MOESM3_ESM.tif]

## Slide 1
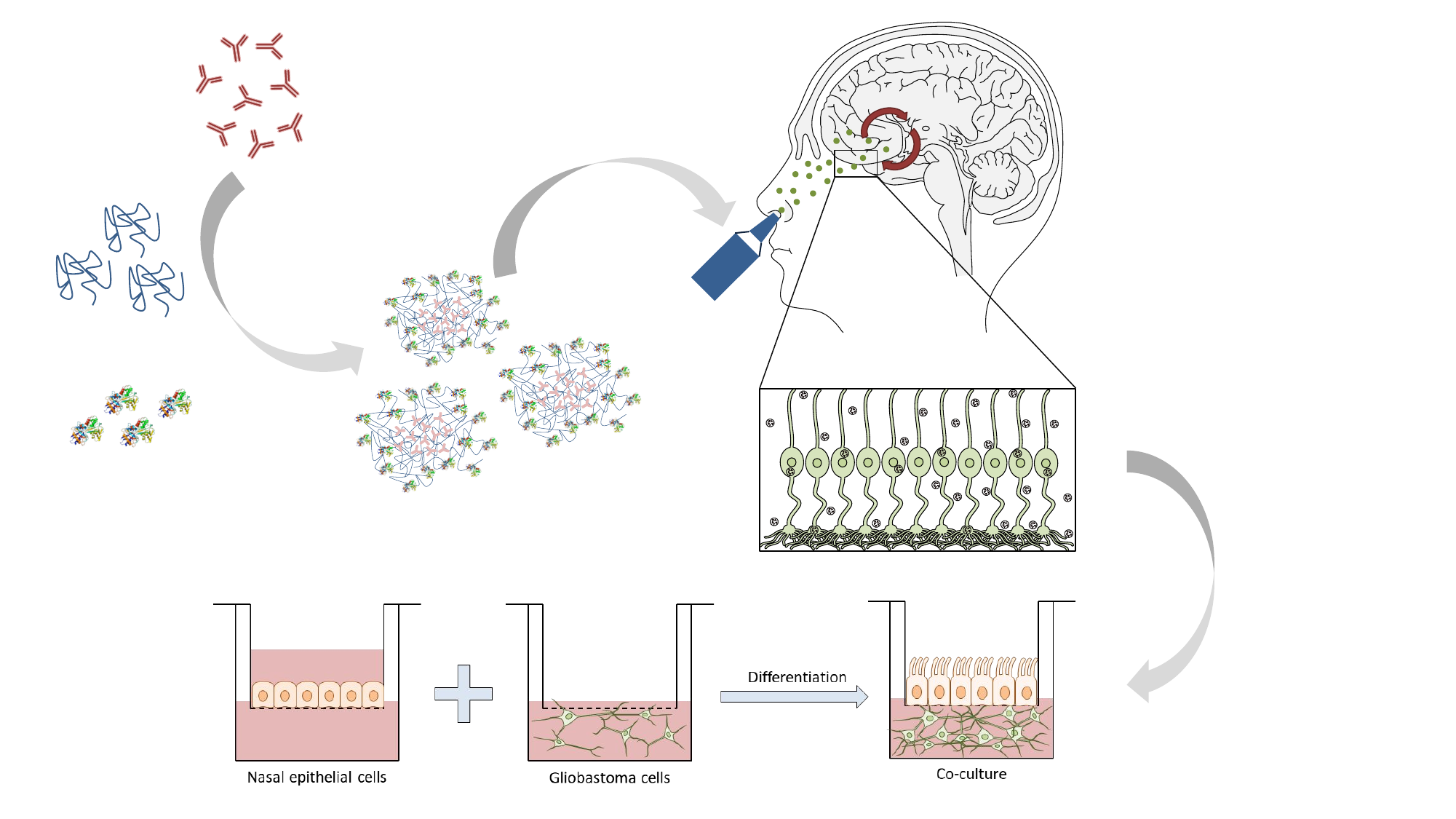

Supplement: Supplementary file 4 — Supplementary file4 (PPTX 278 KB) [file 13346_2022_1245_MOESM4_ESM.pptx]
